# Using the Haddon matrix to explore medical response strategies for terrorist subway bombings

**DOI:** 10.1186/s40779-019-0209-6

**Published:** 2019-06-14

**Authors:** Tie-Cheng Yan, Min Yu

**Affiliations:** Institute of Health Service and Transfusion Medicine, Academy of Military Medical Sciences, No. 27 Taiping Road, Beijing, 100850 China

**Keywords:** Haddon matrix, Subway, Terrorism, Bomb attack, Medical response

## Abstract

**Background:**

Since the 1970s, terrorist bombings in subways have been frequently occurring worldwide. To cope with this threat and to provide medical response countermeasures, we analyzed the characteristics of subway bombing terrorist attacks and used the Haddon matrix to explore medical response strategies.

**Methods:**

First, we analyzed 111 subway bombings from 1970 to 2017 recorded in the Global Terrorism Database to provide a reference for the strategy exploration. Then, we convened an expert panel to use the Haddon matrix to explore the medical response strategies to subway bombings.

**Results:**

In recent decades, at least one bombing attack occurs every 3 years. Summarized by the Haddon matrix, the influencing factors of medical responses to conventional subway bombings include the adequacy of first-aid kits and the medical evacuation equipment, the traffic conditions affecting the evacuation, the continuity and stability of communication, as well as the factors exclusively attributed to dirty bomb attacks in subways, such as ionizing radiation protection capabilities, the structure of the radiation sickness treatment network based on the subway lines, and the disposal of radioactive sewage. These factors form the basis of the strategy discussion.

**Conclusion:**

Since subway bombings are long-term threats, it is necessary to have proper medical response preparation. Based on the Haddon matrix, we explored the medical response strategies for terrorist subway bombings, especially dirty bomb attacks. Haddon matrix can help policymakers systematically find the most important factors, which makes the preparations of the response more efficient.

## Background

Since the 1970s, the global occurrence of subway terrorist bombings has been increasing, especially in the twenty-first century. Several attacks have occurred in London (2005), Moscow (2010), Minsk (2011), Brussels (2016) and St. Petersburg (2017), which have shocked the world, causing mass casualties while seriously destroying societies’ stability [1]. Subway bombings have become a fixed mode of terrorist attacks and may occur at unpredictable times and places in the future. It is worth assessing the emergence response preparedness of every country that has a subway system. Subway bombings always paralyze the urban rail transit system, and successful bombings will lead to a large number of casualties in a short amount of time, which requires a rapid medical response. Medical response is an integral part of antiterrorism campaigns, which reduces the damage to the public and society caused by terrorist bombings. Studies on the medical response to subway bombings are mainly based on the 7 July 2005 London bombings for representativeness [2–7], which caused the largest number of casualties: in total, there were 775 casualties and 56 deaths [2]. The studies reported the prehospital and in-hospital responses to the incident [2–4], as well as the characteristics of injuries [2, 5–7]. A report from the government of London provided a detailed analysis of the inadequacies of the medical response [8]. Thereafter, studies aimed at improving medical response capabilities for subway bombings were not often reported.

To cope with this threat and to improve the medical response preparedness capabilities, in this study, we used the Haddon matrix to identify the influencing factors and propose a wider range of medical response strategies for subway terrorist bombings, which can assist in decision-making by policymakers. We also considered the possible occurrences of subway dirty bomb attacks and applied the Haddon matrix to explore exceptional medical response strategies. Since a dirty bomb is made of radioactive materials mixed with explosives, the explosion could have a similar lethality to that of an ordinary bomb and produce radioactive pollutants that have ionizing radiation effect on the human body, which increases public fear [9]. At present, fortunately, no dirty bomb attacks have been reported, but the future possibility of such an attack should not be ruled out. To obtain a thorough understanding of subway bombings, we retrieved data from the Global Terrorism Database (GTD) and selected data on global subway bombing to analyze the characteristics of the attacks and the casualties.

## Methods

### GTD data analysis

It is necessary to obtain a thorough understanding of subway bombings before the Haddon matrix analysis. “Subway”, “Metro”, “Underground” and “Tube” are usually used to denote an underground transportation system, so these four terms were selected for searching the worldwide data on subway bombings from 1970 to 2017 in the GTD, and 2850 records were retrieved. Each of the retrieved records was screened using two attributes, namely, “Target Type” and “Type of Attack”, which were “Transportation” and “Bombing/Explosion,” respectively. Then, the records conforming to these two attributes were included for the analysis by reading the “Incident Summary” and the “Target Information” of each record. Finally, 111 records out of 2850 were selected.

Medians and percentages were used to describe the characteristics of the subway terrorist bombing attacks and the casualties. A histogram was employed to demonstrate the temporal distribution of subway bombing attacks on different continents. A correlation analysis was used to analyze the correlation between the number of fatalities and the number of injured victims per year. The data were analyzed using Microsoft Office Excel 2007(Microsoft Corporation, Redmond, WA, USA) and R 3.1.0(R Foundation for Statistical Computing, Vienna, Austria). The analysis results provide a reference for an influencing factor analysis and strategic exploration using the Haddon matrix.

### The Haddon matrix

In the 1970s, Dr. William Haddon, Jr., a leader in highway-accident research and prevention in the US, developed the Haddon matrix, which was used for brainstorming traffic accidents’ influencing factors and potential preventive strategies. The Haddon matrix is a conceptual framework with three rows and four columns: the phases in the traffic crash and injury process, namely, precrash, crash, and postcrash phases, define the three rows of the Haddon matrix [10]. The four columns of the Haddon matrix are defined by the following four factors: host, agent/vehicle, physical environment, and social environment/organizational culture. The Haddon Matrix is used as a model for the analysis of influencing factors; the intervention for each factor in the analysis matrix can reduce the possible occurrences of traffic-related injuries (Table 1). The Haddon matrix effectively deepened the understanding of several traffic-related injury factors, such as a person’s behaviour, their vehicle, and the road condition. Furthermore, this matrix provided relevant departments with strong support for controlling traffic injuries in a comprehensive and systematic way. On the basis of the analysis of the various factors in the Haddon matrix, the countermeasures to prevent traffic injuries can be formulated by considering the following aspects: 1) reduce the personnel exposure to risk factors; 2) prevent the occurrence of traffic accidents; 3) reduce the injury severity from the accident; and 4) reduce the number of patients by improving treatment methods after the accident.

**Table 1:** The Haddon matrix and traffic accidents

Phase	Host	Agent/Vehicle	Physical environment	Social environment/Organizational culture
Pre-crash	•Information •Attitude •Traffic police officers’ law enforcement efforts •...	•Lighting •Brakes •Vehicle performance •...	•Road design and layout •Inadequate signage •...	•Speed limits •...
Crash	•Use of fixtures •Degree of traffic crash injuries •...	•Role of fixtures •Anti-collision design •...	•Collision-protection facilities on the road •...	•...
Post-crash	•Organization of the medical response •First-aid level •...	•Risk of a vehicle fire •Difficulties when entering and exiting the vehicle •...	•Suitable rescue equipment •Traffic congestion •...	•Assistance from family and society •Insurance •...

…: The matrix presented here only serves as an example, “…” indicates the remaining factors

Subsequently, the application scope of the Haddon matrix has been expanded. Researchers have used the Haddon matrix to study public health emergency preparedness and the prevention of sports injuries and injuries in other situations [11–13]. The Haddon matrix can also be used by the administrative staff and researchers engaged in terrorist attack prevention and control. Moreover, this matrix can provide a comprehensive and systematic analytical framework for the formulation of medical response strategies for terrorist attacks.

### Convening the expert panel

An expert panel was convened in June 2018 to analyze the influencing factors of medical response to subway bombings using the Haddon matrix. We identified nine experts from the Academy of Military Medical Sciences to serve on the expert panel, including two emergency management experts, two emergency nuclear medicine experts, an expert in anti-chemical weapons research, two epidemiologists engaged in infectious disease control and two emergency medical responders; all of these experts had practical CBRNE medical response experience. A seminar held at the Academy was attended by the expert panel, the moderator first introduced the Haddon matrix and the results of GTD data analysis to the experts and provided them the seminar purpose and the background of subway terrorist bombings. Then, the experts collectively discussed the influencing factors and the moderator recorded them in the Haddon matrix. The inclusion of each influencing factor was subject to the approval of more than half of the experts. Based on the brainstorming method, experts analyzed the influencing factors from three main aspects: the terrorists and the medical response staff, the hazardous materials and the environment that affects the response, with three phases (pre-event, event, and post-event). The event phase was defined as the duration from the explosion to the end of the medical response at the site.

## Results

### GTD data analysis results

In the GTD, the success of a terrorist attack is defined on the basis of the tangible effects of the attack. For instance, a typical successful bombing is one in which the bomb detonates and destroys property and/or kills individuals or when a bomb explodes inside a building. Of the 111 subway bombing attacks, 77.5% (86 of 111) were successful, 22.5% (25 of 111) were unsuccessful; 8.1% (9 of 111) were suicide attacks, and 91.9% (102 of 111) were not suicide attacks. A bombing occurred at least every 3 years.

**Fig. 1:**
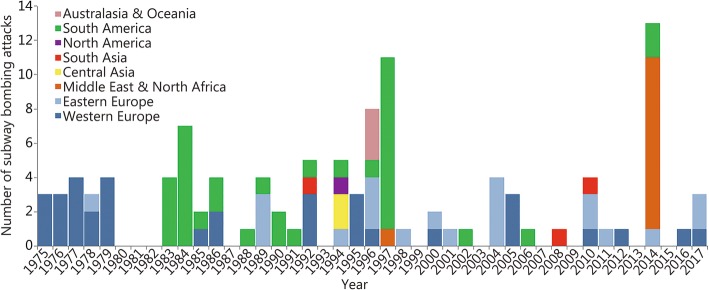
The time distribution of the subway bombing attacks in eight regions of the world (1975–2017). Each bar shows the cumulative number of attacks per year in different regions. The number includes both successful and unsuccessful attacks

The annual maximum number of bombing attacks was 13 in 2014, and the median number was 2 (using the Shapiro-Wilk normality test: *W* = 0.798, *P* < 0.001). More than five attacks were recorded in the following countries: Chile (21), Russia (15), Venezuela (11), Egypt (10), Spain (10), Great Britain (9), and France (6). Europe and South America were two hardest-hit areas, especially Europe, where attacks have occurred most frequently in the past 20 years (Fig. 1).

**Fig. 2:**
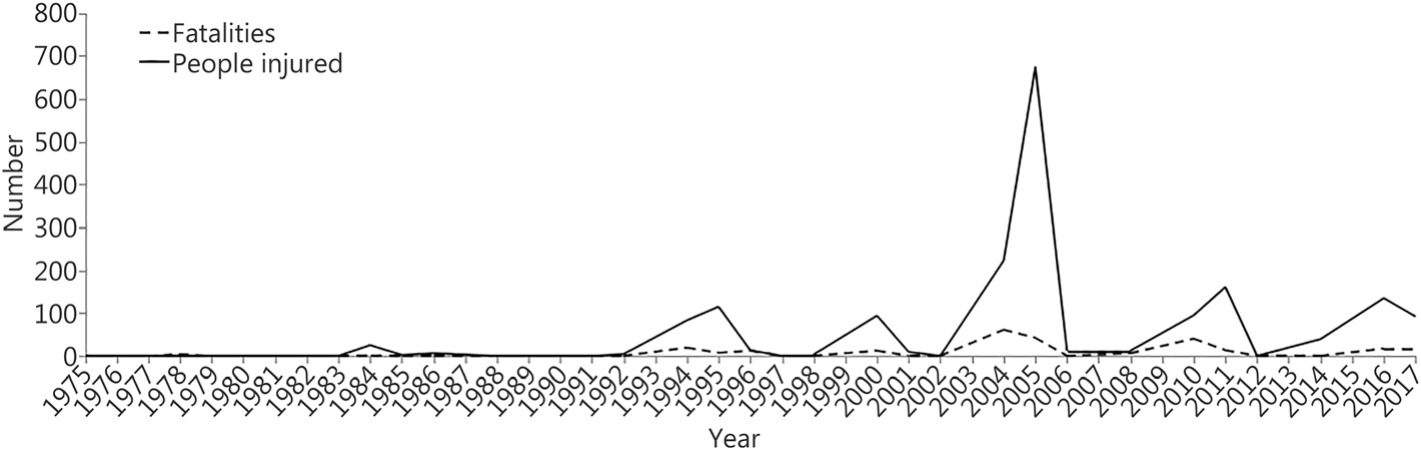
The time distribution of the number of fatalities and the number of injured victims caused by the subway bombing attacks (1975–2017)*.* A positive correlation between the number of fatalities and injured victims in the same period was observed (*r* = 0.748, *P* < 0.001)

Twelve records, which showed an “unknown” number of fatalities or injured victims, were not suitable for further analysis. Of the remaining 99 records, 20.2% (20 of 99) indicated that fatalities occurred in the attacks, and 36.4% (36 of 99) indicated that injuries occurred. Between 1970 and 2017, the first subway bomb attack occurred in 1975, and the total number of fatalities and injured victims were 250 and 1797, respectively. The records showed that suicide subway bombings first occurred in 2004, and the number of fatalities and injured victims caused by all the suicide attacks accounted for 54.4% (136 of 250) and 59.4% (1068 of 1797) of the total incidents, respectively. As shown in Fig. 2, three subway explosions in the 2005 London bombings caused the largest number of casualties since 1975, with a total of 716 (42 fatalities and 674 injured victims).

### The analysis results using the Haddon matrix

In this section, terrorist bombing attacks include conventional and dirty bomb attacks. Tables 2 and 3 show the aggregation of the experts’ analysis results. Table 2 shows the influencing factors for the medical response to subway conventional bomb attacks, while Table 3 shows the factors exclusively attributed to the dirty bomb attacks, which are in addition to those factors listed in Table 2.

**Table 2:** The Haddon matrix and the medical response to conventional bomb attacks in subway systems

Phase	Host	Agent/Vehicle	Physical environment	Social environment/Organizational culture
Pre-event	•Adequacy of the medical and paramedical staff •Standards of individuals’ training •Mental preparation for the response to the attacks •Malicious determination and abilities of the terrorists	•Sources of the explosives	•Adequacy and effectiveness of first-aid kits and instruments •Adequacy of the medical evacuation equipment •Distribution of hospitals along the subway lines •The subway design for passengers’ emergency evacuation	•Construction level of emergency command system for subway antiterrorism •Implementation of subway security inspections •Disaster emergency response preparedness for the subway system •Relevant laws and regulations •Budget
Event	•Suicide or nonsuicide attack •Promptness of the emergency response •First-aid capabilities at the scene •Quality of command, coordination, and control at the scene •The number of people exposed to the terrorist attack threat	•Power of explosion •Secondary damage caused by the destruction of the subway station structures	•Working conditions at the scene •Continuity and stability of communication •Traffic conditions affecting the evacuation	•The public’s awareness of mutual rescue •Implementation of the emergency command system for subway antiterrorism
Post-event	•Health care for the staff •Follow-up treatment for injured persons •Evaluation quality of the medical response	•Residual quantity of harmful gases produced by the explosion in the subway	•Maintenance of first-aid kits, supplements and instruments •Maintenance of the evacuation equipment	•Psychological counseling needs of the public •Post-event media coverage

**Table 3:** The Haddon matrix and the medical response to dirty bomb attacks in subway systems (excluding the content described in Table 2)

Phase	Host	Agent/vehicle	Physical environment	Social environment/Organizational culture
Pre-event	•Standards of individuals’ training, especially for nuclear and radiological attacks •Malicious determination and ability of terrorists	•Sources of radioactive materials	•Adequacy and effectiveness of first-aid kits, instruments, and PPE •Distribution of hospitals along the subway lines (including hospitals that treat radiation sickness) •Adequacy of the medical evacuation equipment for radiation-contaminated casualties •Adequacy and effectiveness of equipment in radiation the detection and decontamination	•None
Event	•Protection capability from ionizing radiation	•The dose and types of radioactive materials released	•Construction of a decontamination station •Water supply for decontamination •Disposal of radioactive sewage	•None
Post-event	•Health care for the staff (including radiation dose detection)	•Duration of radioactive contamination	•Medical evacuation equipment decontamination •Hospital decontamination	•None

*PPE* Personal protection equipment

## Discussion

Medical response strategies were discussed based on the control of the factors in the 12 cells, which were formed from four aspects and three phases in the Haddon matrix. In the following discussion, the main strategies were proposed according to the possibility of implementation.

### The medical response strategies to subway conventional bomb attacks

The strategies to subway conventional bomb attacks were concluded in three phases: the pre-event, event, and post-event phases.

#### Pre-event

##### Preparation of skilled on-site medical and paramedical staff and adequate first-aid kits and equipment

On the basis of the most serious terrorist attack, adequate on-site medical staff, first-aid kits, and equipment must be prepared. The analysis of GTD data shows that suicide subway bomb attacks have become a new trend since 2004, causing far more casualties than nonsuicide attacks. The 2005 London bombings were not only suicide bombings but also serial. The three subway and one double-decker bus explosions left approximately 700 casualties, 56 of them were fatalities, and at least 100 ambulances and more than 250 professionals were needed for the medical response [3].

##### Implementation of training and drills

Professional skill training should be conducted in accordance with an annual plan, and medical response drills for subway bombing attacks should be regularly incorporated into the overall antiterrorism and emergency preparedness plans. The medical response team should emphasize sharing intelligence liaising and cooperating with other emergency response teams. It is advisable to summarize experience through collecting data on training and drills as well as quantitatively and qualitatively analyzing the results. Although London emergency services already had intensive preparation before the 2005 bombings, London still encountered some unprecedented difficulties in the medical response when it happened. Therefore, foreseeing possible threats is essential for training and drills.

#### Event

##### Smooth communication support during the medical response

When a disaster suddenly strikes, such as the 2005 London bombings or the Wenchuan 8-magnitude earthquake on May 12, 2008, in China, the local telephone networks are rapidly overloaded, resulting in the collapse of local mobile communications. However, smooth communication is very important for medical response teams to perform on-site rescues, and accurate and timely information is essential for making the right decisions [14]. At the scene, a public address system and an intercom for bidirectional emergency communications may be an unavoidable option if there are no better means for fixing the overloaded telephone networks [15].

##### Consideration of traffic jams when evacuating casualties

Road congestion is likely to occur and affect the medical response if the attacks occur during rush hour. Furthermore, serial explosions could lead to a large number of casualties. In this case, helicopters and bicycles can become effective modes of transportation. In addition, obtaining effective traffic guidance is very important.

#### Post-event

##### Psychological protection for the emergency staff and the public

Terrorist attacks may cause indelible psychological trauma, especially posttraumatic stress disorder [16, 17], which can sometimes seriously harm people’s lives. Psychological professionals should assess the mental health needs of the staff and the public after the attacks and take effective measures to alleviate psychological harm [18].

##### Evaluation of the medical response

An effective method should be used to evaluate and improve the timeliness and performance of the medical response. The evaluation can also verify the effectiveness of the emergency preparedness plan. A standardized incident report has been advocated to improve the medical response to future incidents. A set of key variables for major incident medical management and a reporting template had been developed [19], which are well worth learning. The accumulation of data, reported in a similarly standardized fashion, would enable the comparison and reporting of series, improving our understanding regarding the optimal medical response [20].

Subway bombings are a long-term threat. The analysis of the GTD data showed that the occurrence frequency of subway bombings has been at least one every 3 years since 1975. Thus, we can fully predict the recurrence of future subway bombings, but predicting the location, scope, and severity is difficult. Although not all attacks have been similar to the London bombings in 2005, which led to massive casualties, medical response preparedness should still be the first option. Moreover, the implementation of medical response drills is an important strategy because it tests the response capabilities in conditions that are close to a real emergency, and the effectiveness of the key factors can be analyzed by the Haddon matrix. Our analysis of the GTD data shows that although suicide subway bombing attacks have occurred only nine times since 2004, they have caused more than half of the total casualties for those years. Therefore, special focus on suicide attacks should be paid in medical response drills.

### The exceptional medical response strategies to subway dirty bomb attacks

Based on the analysis of the factors in Table 3, the main medical response strategies for subway dirty bomb attacks were determined, which are different from those for conventional bomb attacks.

#### Pre-event

##### Preparation of the equipment for responding to a nuclear or radiological incident

Dirty bomb explosions can cause radioactive contamination, which is the major difference from conventional bomb explosions; therefore, we should prepare sufficient personal protective equipment, radiation detection instruments, decontamination equipment, and evacuation vehicles before the attacks. In particular, utilization of evacuation vehicles may be remarkably slowed in a radioactive environment, thereby reducing the overall response efficiency. These vehicles may be contaminated by the radiation from the evacuated casualties; hence, these vehicles are not suitable for the next evacuation task until they have been decontaminated. Therefore, many evacuation vehicles should be prepared.

##### Structure of the radiation sickness treatment network based on the subway lines

The hospitals, especially for radiation sickness treatment, are limited to those in one city; if a large number of contaminated casualties occur in a short amount of time, accomplishing the treatment task may be difficult. Moreover, those hospitals that are far from the explosion site should not be the destination for evacuation. Therefore, regular hospitals should be prepared to serve many casualties, including those suffering from radioactive contamination. Consequently, on the basis of the urban subway layout, the administration should consider designating several hospitals between subway lines as the emergency hospitals and guide the improvement of the hospitals’ emergency plans and supervise their implementation. In this way, the layout of the hospitals can establish a support network for the medical response to a subway dirty bomb attack, maximizing the efficiency of treatment of all the casualties.

#### Event

##### Decontamination of the casualties exposed to radioactive contamination

A radioactive contamination casualty in a life-threatening situation should be immediately sent to the hospital with first aid support, and decontamination should be conducted in the hospital. Delaying treatment or stabilizing of the casualty to facilitate decontamination may put the casualty at additional risk. One or more decontamination stations should be set up around subway stations for decontamination of casualties who are not in danger of death. The implementation of decontamination requires sufficient staff because the decontamination staff may sweat profusely and use up a large amount of physical energy while wearing personal protection equipment. Moreover, a rotating shift is needed to give the staff sufficient rest.

##### Disposal of radioactive sewage

Radioactive sewage from decontamination stations and hospitals is not permitted to be disposed of at will and must be collected. Radionuclide types and the amounts present in sewage should be examined for a hazard classification based on relevant standards to choose the corresponding radioactive sewage disposal method.

#### Post-event

##### Individual radiation dose test for the staff

After the rescue, the individual radiation dose of the staff should be tested, including external and internal irradiation, and the degree of exposure to ionizing radiation needs to be comprehensively assessed.

Unlike conventional bombs, dirty bombs are characterized by the release of ionizing radiation from its explosion; therefore, all response preparations that are different from those of conventional bombs are performed for radiation decontamination. Personal protection, radiation detection, decontamination, and specific treatments are the main guidelines of the response preparedness plan based on the special consideration of the subway environment. When preparing for subway dirty bomb attack, each hospital should be ready to treat contaminated casualties, especially the hospitals that lie along the subway line.

The Haddon matrix is an effective tool for the strategic exploration of public emergency preparedness and response, which enables policymakers to provide comprehensive and feasible solutions according to various factors. By the Haddon matrix, the medical response strategies were proposed in three phases, namely, the pre-event, event, and post-event phases, which have certain practical significance for coping with the threat of subway bombings.

## Conclusions

In this study, we explored the medical response strategies for subway terrorist bombings by the Haddon matrix. Considering the possibility of dirty bomb attacks in the future, we also concluded that the primary response strategies of subway dirty bomb attacks are different from those of conventional bomb attacks. The analysis of the GTD data in this study provides some background references for strategic explorations.

The three phases and four factor dimensions expressed by the Haddon matrix can help policymakers systematically describe the key factors that affect the medical response to subway bombings, which makes the preparations of the response more efficient. The strategies based on these factors not only provide useful references for the security protection of urban subway systems but also for nuclear security. However, the strategy proposed by using the Haddon matrix is macroscopic, so further study on the details of the medical response implementation is needed. We believe future studies would also benefit from the utilization of the Haddon matrix to analyse factors associated with the medical response implementation.

## Data Availability

The datasets generated and/or analyzed during the current study are available at https://www.start.umd.edu/gtd/

## Abbreviations

CBRNE: Chemical, Biological, Radiological, Nuclear, and Explosive
GTD: Global terrorism database
PPE: Personal protection equipment
